# 

**Published:** 1888-04

**Authors:** 


### CONTENTS, APRIL, 1888 (VOL. III, NO. X).

ORIGINAL ARTICLES.—Management and Treatment of the Insane in Texas—By A. N. Denton, M. D., Austin, Texas .. 409

Treatment of Abortions—By R. S. Gregg, M. D., Manor ... 417

Perinephritic Abscess; Operation, etc.—By R. Menger, M. D., San Antonio, Texas.......... 420

Dysentery; Its Cause and Treatment—By E. M. Bridgeford, M. D., Mexico, Mo.......... 421

Some of the Causes of Failure to cure Chronic Suppuration of the Middle Ear—By J. H. Smith, M. D., Dallas ...... 423

SOCIETY NOTES.—Programme for the Galveston Meeting..... 426

Austin District Medical Society.......... 427

EDITORIAL.—The Coming Meeting; Bury the Hatchet...... 437

Interstate Medical Representation.......... 438

Now's the Time.......... 440

Management of the Insane in Texas—Dr. Denton's Paper.. 441

CULLINGS FROM CONTEMPORARIES.—A Summary of the Contents of Exhanges..........442–447

MEDICAL NEWS AND MISCELLENY.—Comprising numerous items of news of interest to the doctor; marriages, deaths, removals, etc.......... 447

BIBLIOGRAPHY.......... 451

ADVERTISERS NOTICES.......... 455

